# Assessment of psychiatrists’ preparedness in managing disaster-related psychiatric disorders: a survey in Taiwan from post-graduate medical education perspective

**DOI:** 10.3389/fpsyt.2024.1368242

**Published:** 2024-06-06

**Authors:** Po-Chun Lin, Yu-Ching Chou, Lien-Cheng Kao, Fang-Jung Wan, Nian-Sheng Tzeng

**Affiliations:** ^1^ Department of Child Psychiatry, Chang Gung Memorial University Hospital at Linkou, Taoyuan, Taiwan; ^2^ School of Public Health, National Defense Medical Center, Taipei, Taiwan; ^3^ Department of Psychiatry, Tri-Service General Hospital, National Defense Medical Center, Taipei, Taiwan; ^4^ Department of Psychiatry, Tri-Service General Hospital, School of Medicine, National Defense Medical Center, Taipei, Taiwan; ^5^ Student Counseling Center, National Defense Medical Center, Taipei, Taiwan

**Keywords:** disasters, psychiatrists, depression, post-traumatic stress disorder, antidepressants, post-graduate medical education

## Abstract

**Background:**

Disaster-related psychiatric disorders (DRPD) present a significant challenge to mental health professionals, yet there is a notable lack of emphasis on the preparedness of psychiatrists in managing these conditions within post-graduate medical education.

**Methods:**

This study utilized a questionnaire to collect data from psychiatrists, focusing on their prior involvement in managing DRPD, perceived competence, medication preferences, and factors influencing their experiences in handling such disorders. Analysis included distribution and ranking of variables, alongside cross-analysis examining associations between demographic factors (age, gender, hospital levels, years of practice, board certification) and treatment experiences, as well as readiness for in-hospital or outside-hospital mobilization in DRPD management.

**Results:**

One hundred and three Taiwanese psychiatrists participated in the study, with the majority reporting involvement in managing DRPD (71.8%), particularly in post-traumatic stress disorder (PTSD) and depression. Antidepressants, specifically serotonin selective reuptake inhibitors, were commonly preferred for DRPD treatment, including PTSD and depression. Psychiatrists aged over 40, with more than 10 years of practice, and hold the board-certified status, showed greater experiences for outside- or inside- the hospital mobilization in DRPD management.

**Conclusion:**

Findings suggest that within post-graduate medical education, Taiwanese psychiatrists demonstrate significant experience, willingness, and capacity to effectively manage DRPD. However, there is a need to integrate comprehensive training on disaster psychiatry into post-graduate psychiatric education programs to further enhance preparedness and optimize outcomes in managing these challenging conditions.

## Introduction

Disasters, characterized by their unpredictability and capacity for causing death, trauma, and property destruction (1), affect millions globally each year. The frequency and impact of these events are escalating due to climate change and population density growth (2), defining disasters by three key characteristics of large-scale traumatic events: significant harm or fatalities among a large group of people, societal disruption, and ensuing mental and physical health consequences (1, 3, 4).

The aftermath of disasters encompasses cognitive, emotional, and physiological responses, leading to predictable psychiatric morbidity (5, 6). Beyond post-traumatic stress disorder, common mental health effects include distress, grief, anxiety disorders, and depression (5–7), for examples, from the exposure to natural (8) or deliberately caused (9) disasters. Individuals often resort to self-medication, primarily with alcohol, risking harm and substance use disorders while delaying the recognition of mood and anxiety disorders (10).

The demands on psychiatrists differ significantly from routine clinical practice (10). Psychiatrists in hospitals must be informed about disaster plans and engage in planning processes, ensuring patients have crisis plans covering mental health management and accessing post-disaster treatment and medication (11, 12). Rapidly mobilized mental health teams, equipped with specialized disaster mental health skills, play a pivotal role in disaster intervention (13). Psychiatrists, as responders, can lead multidisciplinary teams to organize post-disaster psychiatric care, providing crucial assessments and interventions (14). Hence, acquiring fundamental competency in disaster psychiatry is vital for every psychiatrist.

Post-graduate medical education emphasizes clinical training for healthcare professionals, which focused on the standardized training and, under senior physicians’ guidance, develop independent healthcare skills for medical graduates (15, 16). In Taiwan, psychiatric training also following the principles of post-graduate medical education as aforementioned (17, 18), yet there’s a shortage of training competencies incorporating perspectives from both high- and low-income countries (19). In addition, the training for psychiatric trainee might also influence the psychiatrists’ preparedness (20).

Given the heightened risk for natural disasters in several Asian countries, notably by earthquakes, volcanic eruptions, typhoon, and cyclones (21), scant attention has been directed toward assessing psychiatrists’ readiness in managing disaster-related mental health crises (13). This study aims to examine the readiness of Taiwanese psychiatrists in responding to disaster-related mental health crises. We hypothesize discrepancies among psychiatrists based on training in the post-graduate medical education duration and prior clinical experiences.

## Materials and methods

### Study design and participants

This descriptive study employed an innovative questionnaire to assess Taiwanese psychiatrists’ readiness in managing disaster-related psychiatric disorders (DRPD). We distributed 103 questionnaires among Taiwanese psychiatrists in a cross-sectional approach. Inclusion criteria comprised psychiatrists practicing in Taiwan, completing the questionnaire autonomously, comprehending the study’s objectives, and consenting to their questionnaire’s use in the analysis by providing informed consent. The participants completed the self-administered paper-based the questionnaires.

All participants were exclusively Taiwanese, with no inclusion of a control group. Data pertaining to the general population were sourced from prior studies for subsequent discussions in both civilian (22, 23) and military populations (24–26). The questionnaire collected demographic data, including gender, years of clinical experience in psychiatry, and practice facility type. A 5-level rating scale assessed psychiatrists’ preparedness when encountering disasters, gauging familiarity from high to low across several facets: participation in DRPD management, familiarity with disaster-related psychotherapy, acquaintance with medications for DRPD (e.g., PTSD, depression, anxiety, and sleep disorders), and willingness to be mobilized for DRPD relief efforts.

### Statistical analysis

This study employed SPSS statistical software for documentation and analysis. Continuous variables were described using mean and standard deviation. Categorical variables were presented as frequency and percentage. Subjective perceptions of psychiatric drugs and psychotherapy for DRPD. Psychiatrists’ emergency response to DRPD, long-term follow-up willingness, and mobilization readiness were documented using mean and standard deviation. Cross-analysis explored demographic associations with psychotropic medications or psychotherapy, long-term follow-up, and willingness to participate. T-tests, chi-square tests, variance analysis, correlation, regression, and other statistical methods were employed for verification and inference, with statistical significance set at p < 0.05. Multiple linear regression was also used in the assessment of the relationship between factors in unison psychiatrists’ preferences and experiences, with statistical significance set at p < 0.05.

## Results

A total of 103 psychiatrists participated in this study, with male participants, board-certified professionals, and those working in regional hospitals or medical centers comprising the majority (**Table 1**). Notably, 82.5% of these psychiatrists expressed varying degrees of intent to treat, follow up, and even be deployed for the management of DRPD within the scope of this investigation.

**Table 1:** Demography of participants (n=103).

Variants		n.	%
Age (Mean ± SD)	37.90 ± 9.18		
Gender	Male	78	77.2
	Female	23	22.8
	Missing data	2	
Board-certified	Yes	61	63.5
	No	35	36.5
	Missing data	7	
Years of service (Mean ± SD)	10.39 ± 8.62		
Workplace	Medical Center	50	48.5
	Regional Hospital	29	28.2
	Local Hospital	7	6.8
	Clinics	14	13.6
	Others	3	2.9

SD, Standard deviation.

Among these psychiatrists, a significant majority—ranging from 74% to 85%—reported previous experience in treating various forms of DRPD, including PTSD, depressive disorders, anxiety disorders, sleep disorders, and other conditions such as conversion disorders, dissociative disorders, and adjustment disorders. However, only 24% had prior experience in external deployment for DRPD management, while 40% had experience with internal hospital deployment (**Table 2**).

**Table 2:** Psychiatrists’ previous experiences in the participations in the relief of disaster-related psychiatric disorders (n=103).

Events of participations	n. (%)
Treatment of disaster-related psychiatric disorders	74 (71.8)
Treatment of disaster-posttraumatic stress disorder	85 (82.5)
Treatment of disaster–related depressive disorders	85 (82.5)
Treatment of disaster–related anxiety disorders (except PTSD; missing data=2)	78 (77.2)
Treatment of disaster -related sleep disorders	80 (77.7)
Treatment of disaster -related psychiatric disorders (such as: conversion disorders, dissociative disorders, or adjustment disorders)	72 (69.9)
Deployment outside the hospitals for management of disaster -related psychiatric disorders (missing data=2)	24 (23.8)
Deployment inside the hospitals for management of disaster-related psychiatric disorders (missing data=1)	40 (39.2)

**Table 3** outlines the subjective confidence levels of psychiatrists regarding their competence in managing DRPD through psychotherapy. Across various conditions such as PTSD, depressive disorders, anxiety disorders, sleep disorders, and other related disorders, all participating psychiatrists involved conveyed a moderate level of confidence, in a 5-level rating scale, in their ability to conduct both individual and group therapies.

**Table 3:** Subjective feelings of competence in the management of disaster-related psychiatric disorders by psychotherapy (n=103).

Item	Subjective feelings	Mean (± SD)	Rank
1	Competent in management of disaster-related psychiatric disorders	2.69 (1.04)	7
2	Competent in the diagnosis and management of disaster-related psychiatric disorders	2.71 (0.96)	6
3	Competent in the diagnosis and management of posttraumatic stress disorder	2.95 (1.00)	2
4	Competent in the diagnosis and management of depressive disorders	2.97 (1.04)	1
5	Competent in the diagnosis and management of anxiety disorders	2.95 (1.02)	2
6	Competent in the diagnosis and management of sleep disorders	2.91 (1.09)	4
7	Competent in the diagnosis and management of other disaster-related psychiatric disorders (such as conversion disorders, dissociative disorders, or adjustment disorders)	2.76 (1.07)	5
8	Competent in using the technique individual psychotherapy-psychoanalytic or psychodynamic approach	2.41 (1.04)	9
9	Competent in using the technique individual psychotherapy-cognitive behavioral or rational emotional approach	2.58 (1.04)	8
10	Competent in using the technique individual psychotherapy-other approach (such as mindfulness)	2.17 (1.17)	10
11	Competent in using the technique group psychotherapy-psychoanalytic or psychodynamic approach	1.98 (1.12)	12
12	Competent in using the technique group psychotherapy-cognitive behavioral or rational emotional approach	2.11 (1.14)	11
13	Competent in using the technique group psychotherapy-other approach (such as mindfulness)	1.91 (1.07)	14
14	Competent in using the technique individual psychotherapy-other approach (such as debriefing)	1.92 (1.07)	13

Item 1: Disagree completely=1, Agree slightly=2, Agree=3, Agree very much=4, Agree completely=5.

Item 2–14: Unfamiliar completely=1, Familiar slightly=2, Familiar=3, Familiar very much=4, Familiar completely=5.

Regarding the choice of psychotropic medications for DRPD treatment, the majority of psychiatrists in this study leaned toward Selective Serotonin Reuptake Inhibitors (SSRIs) and benzodiazepines (for anxiolysis or sleep) (**Table 4**). Specifically, when addressing disaster-related PTSD, most preferred sertraline, escitalopram, and fluoxetine as their antidepressant choices (**Table 4**).

Table 4Medications and antidepressants chosen by psychiatrists for PTSD (n=103).

**Table:** 

Medications chosen by psychiatrists for PTSD	n. (%)	Ranking
Antidepressant-SSRI	101 (98.1)	1
Antidepressant-SNRI	69 (67.0)	4
Antidepressant-NDRI	35 (34.0)	9
Antidepressant-NaSSA	43 (41.7)	7
Antidepressant-others	26 (25.2)	11
Anxiolytics-Benzodiazepines	86 (83.5)	2
Anxiolytics-buspiron	34 (33.0)	10
Hypnotics-Benzodiazepines	77 (74.8)	3
Hypnotics-Z-drugs	50 (48.5)	6
Antipsychotics	41 (39.8)	8
Beta-antagonists	62 (60.2)	5
Others	4 (3.9)	12

**Table:** 

Antidepressants chosen by psychiatrists for PTSD	n. (%)	Ranking
Fluoxetine	69 (67.0)	3
Sertraline	79 (76.7)	1
Paroxetine	66 (64.1)	4
Fluvoxamine	38 (36.9)	9
Citalopram	47 (45.6)	8
Escitalopram	74 (71.8)	2
Venlafaxine	58 (56.3)	5
Duloxetine	53 (51.5)	6
Milnacipran	22 (21.4)	12
Meclobemide	13 (12.6)	13
Mirtazapine	52 (50.5)	7
Trazodone	32 (31.1)	10
Agomelatine	24 (23.3)	11
Others	1 (1.0)	14

PTSD, posttraumatic stress disorder.

**Table 5** presents data indicating that SSRIs, Serotonin-Norepinephrine Reuptake Inhibitors (SNRIs), and Z-drugs/benzodiazepines were the most frequently selected psychotropic medications for addressing disaster-related depressive disorders, anxiety disorders, sleep disorders, and other associated conditions.

Table 5Medications chosen by psychiatrists for disaster-related psychiatric disorders (n=103).

**Table:** 

Depressive disorders	n. (%)	Ranking
Antidepressant-SSRI	100 (97.1)	1
Antidepressant-SNRI	82 (79.6)	2
Antidepressant-NDRI	49 (47.6)	
Antidepressant-NaSSA	56 (54.4)	
Antidepressant-others	36 (35.0)	
Anxiolytics-Benzodiazepines	68 (66.0)	3
Anxiolytics-buspirone	28 (27.2)	
Hypnotics-Benzodiazepines	58 (56.3)	
Hypnotics-Z-drugs	49 (47.6)	
Antipsychotics	35 (34.0)	
Beta-antagonists	43 (41.7)	
Others	5 (4.9)

**Table:** 

Anxiety disorders	n. (%)	Ranking
Antidepressant-SSRI	86 (83.5)	1
Antidepressant-SNRI	63 (61.2)	3
Antidepressant-NDRI	16 (15.5)	
Antidepressant-NaSSA	31 (30.4)	
Antidepressant-others	23 (22.3)	
Anxiolytics-Benzodiazepines	82 (79.6)	2
Anxiolytics-buspirone	32 (31.1)	
Hypnotics-Benzodiazepines	58 (56.3)	
Hypnotics-Z-drugs	37 (35.9)	
Antipsychotics	15 (14.6)	
Beta-antagonists	53 (51.5)	
Others	3 (2.9)

**Table:** 

Sleep disorders	n. (%)	Ranking
Antidepressant-SSRI	41 (39.8)	
Antidepressant-SNRI	24 (23.3)	
Antidepressant-NDRI	7 (6.8)	
Antidepressant-NaSSA	31 (30.1)	
Antidepressant-others	19 (18.4)	
Anxiolytics-Benzodiazepines	64 (62.1)	3
Anxiolytics-buspirone	9 (8.7)	
Hypnotics-Benzodiazepines	86 (83.5)	1
Hypnotics-Z-drugs	69 (67.0)	2
Antipsychotics	20 (19.4)	
Beta-antagonists	24 (23.3)	
Others	2 (1.9)

**Table:** 

Others	n. (%)	Ranking
Sleep disorders	83 (80.6)	1
Antidepressant-SSRI	53 (51.5)	3
Antidepressant-SNRI	27 (26.2)	
Antidepressant-NDRI	30 (29.1)	
Antidepressant-NaSSA	26 (25.2)	
Antidepressant-others	65 (63.1)	2
Anxiolytics-Benzodiazepines	22 (21.4)	
Anxiolytics-buspirone	50 (48.5)	
Hypnotics-Benzodiazepines	36 (35.0)	
Hypnotics-Z-drugs	28 (27.2)	
Antipsychotics	31 (30.1)	
Beta-antagonists	4 (3.9)	

**Table 6** illustrates that psychiatrists with longer years of service (10 years or more) and holding board-certified status are associated with greater experience in managing DRPD and demonstrate a preference for specific mobilization locations, whether out of hospital or within hospital settings, for interventions, by the multiple linear regression analysis. Psychiatrists aged 40 or older exhibit more experience in managing DRPD, with statistically significant associations found for DRPD in general, particularly anxiety disorders, and both out-of-hospital and in-hospital intervention mobilizations. There is no discernible association between gender and hospital levels with regard to experience in managing DRPD.

**Table 6:** Factors related to the experiences in dealing with disaster-related psychiatric disorders and the places of mobilization for interventions by multiple linear regression analysis (n=98).

	Any	PTSD	Depression	Anxiety	Sleep disorders	Others	Out of hospital	In the hospital
	aOR (95%)	aOR (95%)	aOR (95%)	aOR (95%)	aOR (95%)	aOR (95%)	aOR (95%)	aOR (95%)
**Age** **(age ≧40 vs <40 y/o)**	5.873(1.775–19.424)**	2.743(0.721–10.436)	3.522(0.836–14.827)	4.363(1.064–17.898)*	3.276(0.898–11.957)	2.458(0.821–7.361)	18.922(3.624–98.789)***	3.706(1.331–10.320)*
**Gender** **(Male vs Female)**	0.947(0.325–2.757)	1.216(0.367–4.028)	0.656(0.186–2.314)	1.313(0.422–4.086)	0.935(0.296–2.957)	2.059(0.721–5.878)	0.462(0.072–2.982)	0.883(0.297–2.619)
**Medical center** **(non-medical center vs medical center)**	0.854(0.312–2.339)	1.133(0.361–3.562)	3.152(0.884–11.235)	3.146(0.980–10.100)	2.308(0.753–7.076)	1.486(0.558–3.957)	1.696(0.499–5.763)	0.580(0.222–1.517)
Years of practice (vs < 10 years)								
**10–19 years**	6.282(1.677–23.531)**	5.354(1.001–28.624)*	10.346(1.205–88.823)*	15.124(1.779–128.592)*	4.520(1.092–18.712)*	3.100(0.905–10.614)	29.723(3.138–281.524)**	4.476(1.419–14.115)*
**≧20 years**	7.692(1.447–40.884)*	2.979(0.541–16.389)	7.295(0.831–64.014)	9.656*1.106–84.326)*	9.570(1.107–82.695)*	4.578(0.876–23.925)	68.086(6.333–732.012)***	3.378(0.936–12.188)
**Board-certified** **(non-board-certified vs Board-certified)**	0.118(0.040–0.347)***	0.047(0.009–0.247)***	0.206(0.062–0.684)**	0.188(0.060–0.590)**	0.121(0.038–0.392)***	0.290(0.109–0.772)*	0(0) (No Out of institute in non-Board-certified)	0.193(0.067–0.560)**

*p < 0.05, ** p< 0.01, *** p< 0.001.

## Discussion

Disasters and public health crises pose multifaceted challenges, emphasizing the pivotal role psychiatrists play in addressing post-disaster psychiatric needs (11, 13, 27). This study represents the first exploration of disaster preparedness among Taiwanese psychiatrists and presents several notable findings. Primarily, a significant majority of these professionals exhibit substantial experience, willingness, and capability in managing DRPD. However, while many have treated DRPD, fewer have been involved in hospital deployments for DRPD management, signaling potential areas for improvement.

In Taiwan’s psychiatric resident training, the clinical training program must contain the Suicide Prevention and Disaster Psychiatry Program (17). However, in the results of this study, the average of the participants’ subjective feelings of competence in the 5-level rating scales are between 1.91–2.97 in the psychotherapeutic methods for DRPD. Among these psychotherapeutic techniques, the lowest feelings of competence are group and individual psychotherapies. In addition, the 3^rd^ rank of medications chosen by psychiatrists for PTSD are benzodiazepine. However, benzodiazepines are better avoided the pharmacotherapy for PTSD (28–30).

In the disaster psychiatric response, the interdisciplinary team is needed (31–34). The post-graduate education about the disaster responses for nurses (35), psychologists/counselors (36, 37), and social workers (38) for disaster-related general and mental health services are also important. Therefore, the core competence in the training psychiatric residents is needed, in the post-graduate medical education.

The study notes a perceived lack of competence in utilizing psychotherapeutic approaches for DRPD. Core competence training in the postgraduate psychiatric education programs might enhance the subjective and objective competence in individual and group psychotherapies (39), even for the DRPD (40).

In this study, the deployment experiences of participants are associated with age, the length of practice, and board-ship in psychiatry. Incorporating real-world deployment experiences into postgraduate training might enhance psychiatrists’ readiness and confidence in disaster response (41, 42).

The variance in reported DRPD prevalence (ranging from 1.5% to 74%) in prior Taiwan-based studies may stem from methodological disparities, differing study populations, diagnostic criteria, and case definitions. This variance could also be attributed to Taiwan experiencing fewer wars and natural disasters compared to other regions. As a result, the populace might initially seek primary care providers over psychiatrists during disasters, highlighting potential gaps in popularizing disaster psychiatry in Taiwan (43).

Recent clinical guidelines underscore the efficacy of SSRIs and SNRIs in managing post-traumatic stress, recommending these medications for eligible patients (44). Complementary studies have also highlighted the benefits of benzodiazepines or nonbenzodiazepine agents for disaster-related anxiety and sleep disorders (45, 46). Correspondingly, the preference of Taiwanese psychiatrists for SSRIs, SNRIs, and Z-drugs/benzodiazepines aligns with international guidelines, indicating their substantial knowledge in managing DRPD through pharmacotherapy.

Psychotherapy’s significance in post-disaster mental health recovery cannot be overstated, especially in providing continuous psychological support (47–50). Despite evidence favoring psychotherapy’s efficacy over medications (51, 52), our study revealed psychiatrists’ perceived lack of competence in utilizing psychotherapeutic approaches for DRPD. This underscores the need for bolstering psychotherapy training among Taiwanese psychiatrists.

### Limitations

This study’s limitations include its cross-sectional nature, hindering causal inference, and the lack of differentiation in the questionnaire regarding special needs among different age groups, such as children and the elderly. Additionally, a low response rate might introduce non-response bias, potentially attributed to various factors like workload and insufficient incentives. Therefore, we admitted that non-response bias existed in this study. One potential way to solve this problem in enhancing post-graduate medical education, since several studies have proved that post-graduate medical education could enhance the for the manpower development and capacity building in academic and research competence (53), workers’ skills in child and adolescent mental health (54), or providing culturally relevant and sensitive psychiatric training (55). Thirdly, in this study, we didn’t conduct the competence of the knowledge in the psychiatrists for the international treatment guidelines for DRPD. Further studies for this competence in the psychiatrists are needed in the future, since evidence-based guidelines for diagnosing and treating DRPD, such as PTSD, serve as valuable tools for psychiatrists and other healthcare professionals, facilitating the formulation of tailored treatment plans for their patients (56).

## Conclusions

The study revealed that over 90% of participants exhibited intent to treat, follow-up, or be involved in the management of DRPD, while nearly 80% possessed prior experience in treating these disorders. Findings suggest that within post-graduate medical education, Taiwanese psychiatrists demonstrate significant experience, willingness, and capacity to effectively manage DRPD. However, there is a need to integrate comprehensive training on disaster psychiatry into post-graduate psychiatric education programs to further enhance preparedness and optimize outcomes in managing these challenging conditions.

## Data availability statement

The raw data supporting the conclusions of this article will be made available by the authors, without undue reservation.

## Ethics statement

This study received approval from the Institutional Review Board at Tri-Service General Hospital, and written informed consent was obtained from all participants (TSGH IRB: 2-104-05-136). The studies were conducted in accordance with the local legislation and institutional requirements. The participants provided their written informed consent to participate in this study.

## Author contributions

P-CL: Data curation, Formal analysis, Funding acquisition, Investigation, Writing – original draft. Y-CC: Data curation, Investigation, Writing – original draft, Methodology, Validation. L-CK: Data curation, Investigation, Methodology, Writing – original draft, Conceptualization, Formal analysis, Resources. F-JW: Conceptualization, Data curation, Investigation, Methodology, Resources, Writing – original draft. N-ST: Conceptualization, Data curation, Formal analysis, Investigation, Methodology, Resources, Writing – original draft, Funding acquisition, Project administration, Supervision, Validation, Writing – review & editing.
